# Functional stabilization and neuromuscular adaptation in active condylar hyperplasia: a comparative cohort study

**DOI:** 10.22514/jofph.2026.066

**Published:** 2026-09-12

**Authors:** Andrea Erica Bono, Mariana Gonzalvo

**Affiliations:** ^1^Faculty of Dentistry, National University of La Plata, 1900 La Plata, Argentina; ^2^Faculty of Dentistry, University of Buenos Aires, 1053 Buenos Aires, Argentina; ^3^Faculty of Dentistry, Catholic University of La Plata, 1900 La Plata, Argentina

**Keywords:** Unilateral condylar hyperplasia, SPECT/CT, Surface electromyography, Orthopedic stabilization, Temporomandibular joint, Mandibular asymmetry

## Abstract

**Background**: Active unilateral condylar hyperplasia (UCH) is a progressive mandibular growth disorder characterized by facial asymmetry, occlusal alteration, and mandibular deviation. Although single-photon emission computed tomography/computed tomography (SPECT/CT) is widely used to assess condylar hyperactivity, little is known about the clinical and neuromuscular adaptations occurring during the observation period before definitive treatment. This study evaluated whether conservative functional stabilization is associated with adaptive clinical and neuromuscular changes during this period. **Methods**: This prospective comparative observational cohort study included 49 patients with active unilateral condylar hyperplasia confirmed by SPECT/CT. Thirty-seven patients received full-time orthopedic stabilization with a rigid mandibular intraoral orthosis, whereas 12 were managed by observation alone. Clinical outcomes included pain intensity, maximum mouth opening, and mandibular midline deviation. Surface electromyography (sEMG) was performed at baseline and after approximately 3 months. SPECT/CT examinations were obtained at baseline and follow-up (mean, approximately 14 months). Within-group and between-group comparisons, effect sizes, percentage changes, and 95% confidence intervals were calculated. **Results**: Orthopedic stabilization significantly improved mandibular midline deviation, maximum mouth opening, and neuromuscular function. Composite resting sEMG activity decreased, whereas functional muscle recruitment increased in the treated cohort. In contrast, the observation cohort demonstrated increased resting muscle activity with minimal functional changes. Significant between-group differences were observed for resting and functional sEMG outcomes. SPECT/CT metabolic changes were also detected, but were less consistent and clinically informative than the documented clinical and neuromuscular adaptations. **Conclusions**: Full-time orthopedic stabilization was associated with measurable clinical and neuromuscular adaptation during the observation period preceding definitive treatment. These findings support functional stabilization as a conservative approach that may promote a more favorable neuromuscular and biomechanical environment while treatment decisions are being made. SPECT/CT findings should be interpreted as complementary semiquantitative observations rather than definitive indicators of growth modification.

## 1. Introduction

Active unilateral condylar hyperplasia (UCH) is an uncommon mandibular growth disorder characterized by progressive facial asymmetry, occlusal alteration, and mandibular deviation. Historically, it has been regarded primarily as a localized disorder of the mandibular condyle; however, contemporary evidence indicates that its clinical phenotype is heterogeneous and that its pathogenesis remains incompletely understood. Genetic, hormonal, vascular, inflammatory, traumatic, and mechanical factors have all been proposed as potential contributors to the disorder [1, 2, 3, 4, 5, 6].

The classical classification proposed by Obwegeser and Makek distinguishes hemimandibular hyperplasia, hemimandibular elongation, and mixed forms [1]. Wolford and colleagues subsequently proposed a broader classification system incorporating clinical, imaging, and histopathological characteristics, together with conditions that may mimic or contribute to condylar enlargement [2]. This phenotypic diversity is clinically relevant because vertical, horizontal, and mixed growth patterns may result in distinct occlusal, muscular, and temporomandibular joint loading conditions [2, 3, 7, 8].

Diagnosis is based on clinical progression, facial and intraoral examination, imaging findings, and nuclear medicine assessment. Single-photon emission computed tomography/computed tomography (SPECT/CT) is widely used to identify relative condylar hyperactivity; however, the technique remains semiquantitative and should be interpreted in conjunction with clinical findings, growth status, and serial documentation [7, 8, 9, 10]. Although thresholds based on side-to-side condylar uptake differences have been incorporated into clinical practice, nuclear imaging should not be interpreted as a stand-alone indicator of biological growth arrest or therapeutic success [7, 8, 9, 10].

Conservative management, high condylectomy, orthognathic surgery, or combined surgical-orthodontic approaches may all be appropriate depending on growth activity, patient age, skeletal pattern, severity of asymmetry, occlusal compromise, symptoms, patient expectations, interdisciplinary judgment, and current clinical practice recommendations [5, 6, 11, 12, 13]. Accordingly, high condylectomy should not be regarded as the universal treatment of choice, and the timing of definitive intervention should be determined through comprehensive clinical and functional assessment.

During the interval between diagnosis and definitive treatment planning, patients may experience progressive mandibular asymmetry, unstable occlusal support, altered muscle activity, limited mouth opening, patient-reported symptoms, and asymmetric loading of both temporomandibular joints. Although condylar hyperplasia is not classified as a conventional temporomandibular disorder, contemporary orofacial pain and temporomandibular disorder frameworks advocate diagnosis-driven, individualized, conservative, and reversible management before irreversible therapeutic interventions are considered [13, 14]. Consequently, this observation period represents a clinically important phase during which functional adaptation may occur while definitive treatment is being planned.

Full-time orthopedic intraoral appliance therapy may be considered a temporary functional stabilization strategy during this observation period. Its objective is not to suppress condylar growth biologically, but rather to provide more stable occlusal support, reduce mandibular deviation, improve neuromuscular function, and potentially modulate the functional loading environment. Evidence supporting intraoral appliances in patients with temporomandibular disorders cannot be directly extrapolated to active condylar hyperplasia because the underlying pathophysiological mechanisms differ substantially. Nevertheless, both conditions may involve secondary functional disturbances of the stomatognathic system, including altered occlusal stability, asymmetric mandibular positioning, changes in muscle recruitment, and modified temporomandibular joint loading. Conservative orthopedic appliances are intended to address these functional disturbances through reversible occlusal stabilization rather than by modifying the underlying disease process. Accordingly, although they are not expected to alter the biological activity of condylar growth, they may facilitate functional adaptation during the observation period preceding definitive treatment. Therefore, although indirect, the evidence supporting conservative management of temporomandibular disorders provides a cautious conceptual framework for investigating functional stabilization in this specific patient population rather than direct evidence of treatment efficacy [15, 16, 17, 18, 19, 20].

The primary objective of the present study was to evaluate whether full-time orthopedic functional stabilization is associated with measurable clinical and neuromuscular adaptation in patients with active unilateral condylar hyperplasia during the observation period preceding definitive treatment. A secondary objective was to characterize the accompanying semiquantitative SPECT/CT metabolic changes observed during follow-up.

We hypothesized that functional stabilization may contribute to functional adaptation within the stomatognathic system and potentially to a more favorable biomechanical environment. This proposed mechanism remains biologically plausible, but has not yet been established.

## 2. Materials and methods

### 2.1 Study design

This study was designed as a prospective comparative observational cohort study, reported according to Strengthening the Reporting of Observational Studies in Epidemiology (STROBE) recommendations for observational clinical research [21]. It included a treated cohort receiving full-time orthopedic intraoral appliance therapy and an observational cohort managed without orthopedic intervention. Because treatment allocation reflected routine clinical decision-making rather than randomization, the study should be interpreted as hypothesis-generating rather than as a randomized controlled trial. Patient flow is illustrated in Fig. 1.

**Fig. 1.** Study flow and analytic cohorts. Of the 51 eligible patients initially recruited, 39 entered the orthopedic stabilization cohort and 12 entered the observation cohort. Two patients in the orthopedic stabilization cohort discontinued treatment, resulting in a final analytic cohort of 49 patients: 37 in the orthosis cohort and 12 in the observation cohort. Paired sEMG data were available for 36 and 12 patients, respectively.

### 2.2 Participants

A total of 51 consecutive eligible patients were recruited between January 2018 and December 2024. Diagnosis was established on the basis of progressive clinical findings consistent with active unilateral condylar hyperplasia, including facial asymmetry, mandibular midline deviation, and occlusal changes compatible with unilateral mandibular overgrowth, together with SPECT/CT findings demonstrating increased condylar metabolic activity. Baseline demographic and clinical characteristics of both cohorts are summarized in Table 1.

**Table 1.** Baseline demographic, clinical, electromyographic, and SPECT/CT characteristics of the orthosis and observation cohorts.

Variable		Orthosis cohort (n = 37)	Observation cohort (n = 12)	*p* value
Demographic characteristics				
	Age (yr), mean ± SD¹	28.1 ± 7.0	30.1 ± 7.5	0.430*
	Sex, n (%)			
	• Female	21 (56.8)	10 (83.3)	0.100^†^
	• Male	16 (43.2)	2 (16.7)	
Baseline disease status				
	Affected side, n (%)			
	• Right	15 (40.5)	7 (58.3)	0.340^†^
	• Left	22 (59.5)	5 (41.7)	
Clinical characteristics				
	Maximum mouth opening (mm), mean ± SD	34.95 ± 2.45	34.92 ± 1.31	0.962*
	Mandibular midline deviation (mm), mean ± SD	3.54 ± 1.59	4.17 ± 1.47	0.334*
Neuromuscular characteristics				
	Composite resting sEMG (µV), mean ± SD¹	4.18 ± 0.69	4.00 ± 0.37	0.544*
	Composite maximal voluntary clenching (MVC) sEMG (µV)¹	131.56 ± 6.35	133.45 ± 6.31	0.405*
Imaging characteristics				
	Coronal SPECT/CT uptake ratio, mean ± SD	1.29 ± 0.18	1.41 ± 0.09	0.008*
	Axial SPECT/CT uptake ratio, mean ± SD	1.26 ± 0.33	1.22 ± 0.10	0.556*

Values are presented as mean ± standard deviation (SD) or number (percentage), as appropriate. All variables correspond to the baseline evaluation, performed before initiation of orthopedic stabilization in the orthosis cohort and before the observation period in the observation cohort. Baseline comparisons were performed to assess the initial comparability of the two cohorts. Continuous variables were compared using the Mann-Whitney U test, whereas categorical variables were compared using Fisher’s exact test. Statistical significance was set at p < 0.05. SD, standard deviation; sEMG, surface electromyography; SPECT/CT, single-photon emission computed tomography/computed tomography. ¹Age and sEMG data were available for 36 patients in the orthosis cohort and 12 patients in the observation cohort. *Mann-Whitney U test. ^†^Fisher’s exact test. Bold p values indicate statistical significance (p < 0.05).

Thirty-nine patients initially accepted full-time orthopedic stabilization. Two discontinued treatment before study completion, leaving 37 patients for the final treated clinical and SPECT/CT analyses. Twelve patients were managed by observation alone.

### 2.3 Treatment allocation

Treatment allocation was based on routine clinical decision-making rather than randomization. Following the baseline evaluation, patients who accepted full-time orthopedic stabilization initiated treatment with the intraoral orthopedic device.

The observational cohort did not represent healthy controls; rather, it consisted of patients who met the same diagnostic criteria for active unilateral condylar hyperplasia, but remained under standardized clinical and imaging follow-up without orthopedic intervention during the study period. Assignment to observation reflected routine clinical management and included patients in whom definitive surgical treatment had not yet been undertaken because surgical planning remained under evaluation, continued clinical and imaging surveillance (including repeat SPECT/CT when clinically indicated) was considered appropriate, or multidisciplinary consensus regarding the timing of surgery had not yet been reached. Consequently, these patients received neither orthopedic stabilization nor surgical treatment during the study period.

Therefore, both cohorts fulfilled identical diagnostic criteria at study entry. The principal difference between groups was the clinical management strategy adopted during follow-up rather than baseline disease status. Because treatment allocation reflected individualized clinical decision-making, several patients fulfilled more than one indication for continued observation (*e.g.*, ongoing surgical planning, multidisciplinary reassessment, or serial imaging follow-up). Consequently, the relative frequency of individual allocation reasons was not analyzed separately.

To reduce selection bias, all eligible patients were recruited consecutively using the same diagnostic protocol during the study period, and baseline demographic and clinical characteristics were compared between cohorts (Table 1). Although residual confounding inherent to the non-randomized observational design cannot be excluded, these measures were implemented to enhance comparability between groups. Accordingly, the observation cohort should not be interpreted as a conventional untreated control group, but rather as a clinically observed comparison cohort managed according to routine clinical practice during the period preceding definitive treatment.

### 2.4 Inclusion and exclusion criteria

Inclusion criteria comprised progressive clinical signs consistent with active unilateral condylar hyperplasia, including facial asymmetry, mandibular midline deviation, occlusal discrepancy consistent with unilateral mandibular overgrowth, and SPECT/CT findings demonstrating increased condylar metabolic activity. Exclusion criteria included previous orthognathic or temporomandibular joint surgery, recent facial or cervical trauma, systemic inflammatory disease, fibromyalgia, ongoing orthodontic treatment capable of modifying occlusal contacts, and prosthetic rehabilitation interfering with mandibular registration.

Ethical considerations. The study was conducted in accordance with the principles of the Declaration of Helsinki and was approved by the Ethics Committee of the Faculty of Dentistry, National University of La Plata (Resolution No. 100-000069-19-00). Written informed consent was obtained from all participants or their legal guardians before inclusion in the study. Written consent for publication of clinical photographs was obtained from all patients whose images are included in this manuscript.

### 2.5 Orthopedic appliance

The orthopedic stabilization appliance consisted of a rigid mandibular acrylic splint individually fabricated for each patient. The appliance provided complete and simultaneous occlusal contacts with the opposing maxillary dentition, without posterior disclusion, and incorporated canine guidance during lateral excursions and anterior guidance during protrusive movements.

Mandibular registration was performed following neuromuscular deprogramming using an ultra-low-frequency transcutaneous electrical nerve stimulation (ULF-TENS) device (BioTENS®, BioResearch Associates Inc., Milwaukee, WI, USA). Bilateral stimulation of the motor branches of the trigeminal and facial nerves was delivered using a dual-channel system with self-adhesive surface electrodes positioned over the preauricular regions and the posterior cervical area in accordance with the manufacturer’s protocol. Electrical stimulation was applied at 1 Hz with a pulse duration of 600 μs (two 300-μs phases) for approximately 40–60 minutes until a stable and reproducible neuromuscularly relaxed mandibular position was achieved, as routinely recommended for neuromuscular registration.

The mandibular position obtained following ULF-TENS was recorded using a silicone bite registration material. The vertical dimension of the appliance was determined individually from this neuromuscular position after subtracting approximately 2 mm, corresponding to the physiological interocclusal rest space. Consequently, appliance thickness was individualized rather than predetermined.

### 2.6 Appliance protocol

Patients were instructed to wear the appliance full time (approximately 24 hours per day), including during meals, removing it only for oral hygiene procedures. Monthly follow-up visits were scheduled to verify appliance stability, occlusal contacts, retention, and adaptation. These visits were performed exclusively for appliance monitoring and adjustment, and were not considered outcome assessment time points because the statistical analyses were based on the predefined baseline and follow-up evaluations. Whenever minor loss of adaptation or retention was detected, chairside acrylic relining and selective occlusal adjustments were performed as required to maintain the registered neuromuscular mandibular position.

Patients also received standardized oral hygiene instructions and were advised to report immediately any loss of retention, appliance fracture, instability, or inability to maintain continuous wear. Compliance was assessed clinically at each follow-up visit based on patient interview, appliance condition, and consistency between self-reported wear and clinical findings. All treated patients who completed the study were considered adherent to the prescribed wear protocol.

### 2.7 Adverse events

No serious device-related adverse events were observed during the study. A small number of patients with pre-existing gingival recession reported transient tooth sensitivity during the initial adaptation period, which resolved following minor appliance adjustment and did not require treatment discontinuation. Potential complications associated with inadequate compliance, poor oral hygiene, prolonged interruption of appliance wear, or loss of appliance adaptation were explained to all patients before treatment initiation.

### 2.8 Clinical outcomes

Pain intensity was assessed using a 10-point visual analog scale (VAS), where 0 indicated “no pain” and 10 indicated “the worst imaginable pain”. Maximum mouth opening was measured as the maximum unassisted interincisal distance between the maxillary and mandibular central incisors using a digital caliper and recorded in millimeters. Mandibular midline deviation was measured in maximum intercuspation as the linear distance between the maxillary and mandibular dental midlines using the same digital caliper. Appliance-related discomfort was recorded at each scheduled follow-up visit throughout orthopedic treatment. Clinical outcomes were evaluated as functional and patient-centered measures rather than as evidence of direct modification of condylar growth. All clinical assessments were performed by the same examiner using a standardized protocol.

### 2.9 Frankfurt plane

Surface electromyographic recordings were obtained at baseline and after approximately 3 months using the BioEMG III™ system (BioResearch Associates Inc., Milwaukee, WI, USA). Before electrode placement, the skin was cleaned with 70% isopropyl alcohol to reduce skin impedance. Bipolar self-adhesive surface electrodes (BioFlex EMG Electrodes™, BioResearch Associates Inc., Milwaukee, WI, USA; 40 × 17 mm) were placed bilaterally over the anterior temporalis and masseter muscles according to standardized anatomical landmarks. Electrode placement over the anterior temporalis muscle was determined by palpation during maximal voluntary clenching following the protocol described by Gay *et al*. [22]. Electrodes over the masseter muscle were positioned over the area of greatest muscle bulk, approximately at the midpoint of the line joining the gonial angle and the inferior border of the zygomatic arch. The reference electrode was placed in the submandibular midline region over the soft tissues anterior to the anterior belly of the digastric muscle.

The BioEMG III™ system incorporates a differential analog amplifier with a gain of ×5000 and an input impedance of 10^12^ Ω. According to the manufacturer’s technical specifications, signals were acquired at a sampling frequency of 2000 Hz, with a system bandwidth of 30–1000 Hz, ensuring adequate signal acquisition in accordance with the Nyquist sampling criterion. During signal processing, the electromyographic signal was band-pass filtered between 20 and 500 Hz, and additional digital filtering was performed using the proprietary Noise Buster™ algorithm (BioResearch Associates Inc., Milwaukee, WI, USA) to attenuate 50-Hz power-line interference and its harmonics while preserving the physiological EMG signal.

Patients were seated comfortably with the Frankfurt plane parallel to the floor, eyes open, and the upper limbs resting comfortably.

One standardized recording was obtained for each recording condition after signal stabilization. Recordings were acquired under two standardized conditions: mandibular rest and maximal voluntary clenching (MVC).

During the resting recording, subjects were instructed to maintain the mandible in a relaxed position with the lips slightly apart and without tooth contact for 10 seconds. During MVC, patients were instructed to clench maximally in maximum intercuspation for 10 seconds while avoiding mandibular protrusion or retrusion. Mean electromyographic amplitudes were expressed in microvolts (μV) for each muscle and recording condition [23].

Absolute EMG amplitudes (μV) were used for longitudinal within-subject comparisons. Signal normalization was not performed because the objective of the study was to evaluate changes over time within the same individual rather than comparisons between subjects.

All sEMG recordings were obtained by the same trained examiner using a standardized acquisition protocol to minimize operator-related variability. Electrode placement and recording conditions were standardized for all examinations to maximize within-subject reproducibility.

### 2.10 SPECT assessment

SPECT/CT examinations were performed at baseline and after a mean follow-up interval of approximately 14 months. Following intravenous administration of technetium-99m methylene diphosphonate (99mTc-MDP), image acquisition was performed approximately 2–3 hours after tracer injection according to the institutional nuclear medicine protocol. Image interpretation and semiquantitative analysis were performed by an experienced nuclear medicine radiologist according to the institutional protocol. The radiologist was independent of the research team and interpreted all examinations without knowledge of the patients’ participation in the study, clinical findings, or orthopedic treatment.

Coronal and axial condylar uptake ratios, together with the corresponding relative uptake percentages for each condyle, were recorded as semiquantitative indicators of relative osteoblastic activity.

Regions of interest (ROIs) were manually delineated around each condylar head according to the standardized protocol routinely used by the Nuclear Medicine Department.

A diagnosis of active unilateral condylar hyperplasia was established when progressive clinical findings were accompanied by asymmetric condylar uptake on SPECT/CT. Side-to-side condylar uptake differences ≥10%, in conjunction with progressive clinical findings, were considered consistent with active unilateral condylar hyperplasia. This threshold was not used in isolation. Accordingly, SPECT/CT findings were interpreted together with the overall clinical presentation and were not used as the sole diagnostic criterion.

Because SPECT/CT provides only semiquantitative information, imaging findings were interpreted as indicators of relative osteoblastic activity rather than as direct evidence of current growth velocity or complete biological growth arrest, consistent with contemporary recommendations regarding the interpretation of bone SPECT/SPECT-CT in unilateral condylar hyperplasia. Consequently, uptake ratios approaching the institutional reference range during follow-up were interpreted as indicating lower relative tracer uptake in the previously hyperactive condyle compared with the contralateral condyle at the time of examination, rather than as definitive evidence of biological growth arrest [24, 25, 26, 27].

### 2.11 Statistical analysis

Statistical analyses were performed using Microsoft Excel for Mac (version 16.111.2, Microsoft Corp., Redmond, WA, USA). Continuous variables are presented as mean ± standard deviation (SD), whereas categorical variables are reported as frequencies and percentages.

The distribution of continuous variables was assessed using the Shapiro-Wilk test. Because several variables did not meet the assumption of normality and the sample size was moderate, non-parametric statistical methods were selected.

Within-group longitudinal comparisons (baseline versus follow-up) were performed using the Wilcoxon signed-rank test, whereas comparisons between the treated and observation cohorts were performed using the Mann-Whitney U test. All statistical tests were two-tailed, and statistical significance was established at *p* < 0.05.

Surface electromyographic analyses were performed separately for the anterior temporalis and masseter muscles under resting conditions and during maximal voluntary clenching. Right and left muscle recordings were automatically averaged by the BioEMG III™ acquisition system to obtain a single representative value for each muscle and recording condition, thereby reducing the number of statistical comparisons and improving interpretability. Thus, the composite sEMG value represents the average electromyographic activity of the bilateral anterior temporalis and masseter muscles under each recording condition rather than a derived physiological index.

Because this was an exploratory, hypothesis-generating observational study, no formal adjustment for multiple comparisons was performed. Accordingly, statistically significant findings should be interpreted as exploratory and hypothesis-generating rather than confirmatory, particularly in view of the multiple outcome measures evaluated. Therefore, *p*-values should be interpreted as descriptive rather than confirmatory.

Missing data were handled using complete-case analysis. Consequently, only patients with paired baseline and follow-up measurements were included in the corresponding longitudinal analyses.

## 3. Results

### 3.1 Sample flow and baseline characteristics

Of the 51 eligible patients initially recruited, 39 entered the orthosis cohort and 12 entered the observation cohort. Two patients in the orthosis cohort discontinued treatment, resulting in a final analytic sample of 37 orthosis patients and 12 observation patients (total n = 49).

Thirty-seven patients underwent full-time orthopedic stabilization, whereas 12 patients remained under observation without orthopedic intervention throughout the study period. Clinical and SPECT/CT analyses included all 49 participants. Paired sEMG analyses included 48 participants because one patient in the orthopedic stabilization cohort did not have complete interpretable paired electromyographic recordings.

Overall, the orthopedic stabilization and observation cohorts were comparable at baseline with respect to demographic, clinical, and neuromuscular characteristics. The only statistically significant baseline difference was the coronal SPECT/CT uptake ratio, which was higher in the observation cohort (Table 1).

### 3.2 Clinical outcomes

Clinical outcomes are summarized in Fig. 2.

**Fig. 2.** Clinical outcomes associated with orthopedic stabilization. Clinical changes observed during follow-up in patients with active unilateral condylar hyperplasia. (A) Mean change in maximum mouth opening from baseline to final follow-up. (B) Mean change in mandibular midline deviation. (C) Pain intensity (VAS) during follow-up. (D) Progressive improvement in patient-reported comfort during full-time orthopedic appliance use across eight clinical evaluations. Error bars indicate 95% confidence intervals where applicable. VAS, visual analog scale.

Patients receiving orthopedic stabilization demonstrated significant improvements in maximum mouth opening and mandibular midline deviation during follow-up, whereas no significant changes were observed in the observation cohort (Fig. 2A,B).

The reduction in mandibular midline deviation corresponded to an approximately one-third decrease relative to baseline in the orthopedic stabilization cohort.

Pain intensity progressively decreased throughout follow-up in the orthopedic stabilization cohort, whereas it remained relatively stable in the observation cohort (Fig. 2C).

Patient-reported appliance comfort improved progressively throughout treatment, indicating favorable adaptation to continuous appliance wear (Fig. 2D).

Representative clinical photographs illustrating correction of facial asymmetry, improved mandibular centering, and occlusal stabilization with the orthopedic appliance are presented in Fig. 3. These images illustrate the typical clinical presentation and treatment-related changes and were not included in the quantitative analyses.

**Fig. 3.** Representative clinical effects of orthopedic stabilization. Representative clinical photographs obtained from the same patient before treatment and during full-time orthopedic stabilization. (A) Pretreatment frontal view demonstrating facial asymmetry and mandibular deviation. (B) Frontal view obtained during orthopedic stabilization showing improved mandibular centering. (C) Pretreatment occlusal relationship. (D) Occlusal relationship with the orthopedic appliance in place, illustrating stabilization of mandibular position. These photographs are presented for illustrative purposes only and were not included in the quantitative analyses. Yellow arrows indicate the direction of mandibular deviation and its correction during orthopedic stabilization.

The proportion of participants reporting satisfactory comfort increased progressively from the first to the eighth follow-up visit. Because these data were ordinal and were not collected in the observation cohort, they are presented descriptively in Fig. 2D.

### 3.3 Surface electromyography

Paired surface electromyography (sEMG) analyses included 48 participants (36 in the orthopedic stabilization cohort and 12 in the observation cohort), as one participant in the orthopedic stabilization cohort did not have complete interpretable paired sEMG recordings.

Composite and muscle-specific electromyographic outcomes are presented in Tables 2 and 3, respectively (Fig. 4).

**Table 2.** Composite surface electromyography outcomes at rest and during function.

Outcome	Favorable direction	Group	n	Baseline	3 months	Mean change	Change (%)	95% CI for change	Within-group *p*	Std. effect	Between-group *p*	Cliff delta
Composite resting sEMG (µV)	Decrease	Orthosis	36	4.18 ± 0.69	3.32 ± 0.97	−0.86	−20.6	−1.27 to −0.45	0.001	−0.71	<0.001	−0.70
		Observation	12	4.00 ± 0.37	4.57 ± 0.59	+0.57	+14.2	+0.17 to +0.97	0.016	+0.90		
Composite functional MVC sEMG (µV)	Increase	Orthosis	36	131.56 ± 6.35	158.11 ± 20.85	+26.55	+20.2	+19.01 to +34.08	<0.001	+1.19	<0.001	+0.69
		Observation	12	133.45 ± 6.31	135.61 ± 5.69	+2.15	+1.6	−3.67 to +7.98	0.622	+0.23		

*Composite values were calculated as the average of the right anterior temporalis, left anterior temporalis, right masseter, and left masseter recordings under each recording condition. Values are reported as mean ± standard deviation. sEMG, surface electromyography; MVC, maximal voluntary clenching; CI, confidence interval. Negative change in resting activity and positive change in functional MVC activity were considered favorable. Mean change was calculated as the follow-up value minus the baseline value. Within-group p values were calculated using the Wilcoxon signed-rank test. Between-group p values compare change scores between cohorts using the Mann-Whitney U test. Standardized effect was calculated as the mean paired change divided by the standard deviation of the paired change. Cliff’s delta compares change scores between the orthosis and observation cohorts.*

**Table 3.** Muscle-specific surface electromyography outcomes.

Outcome/muscle		Group	n	Baseline	3 months	Mean change	Change (%)	95% CI for change	Within-group *p*	Std. effect	Between-group *p*	Cliff delta
Resting sEMG (µV)												
	Right temporalis	Orthosis	36	4.32 ± 1.04	3.44 ± 1.15	−0.88	−20.4	−1.26 to −0.50	<0.001	−0.79	<0.001	−0.93
		Observation	12	3.59 ± 0.73	4.77 ± 0.86	+1.18	+32.9	+0.79 to +1.58	<0.001	+1.89		
	Left temporalis	Orthosis	36	4.49 ± 1.12	3.12 ± 1.31	−1.37	−30.6	−1.84 to −0.91	<0.001	−1.00	<0.001	−0.82
		Observation	12	3.95 ± 0.94	4.77 ± 0.86	+0.83	+20.9	+0.06 to +1.59	0.025	+0.69		
	Right masseter	Orthosis	36	3.93 ± 0.72	3.51 ± 1.45	−0.42	−10.7	−1.02 to +0.17	0.142	−0.24	0.543	−0.12
		Observation	12	4.08 ± 0.43	4.02 ± 1.13	−0.07	−1.6	−0.92 to +0.79	0.898	−0.05		
	Left masseter	Orthosis	36	3.98 ± 0.57	3.20 ± 1.34	−0.77	−19.5	−1.33 to −0.22	0.010	−0.47	0.018	−0.46
		Observation	12	4.37 ± 0.54	4.71 ± 0.92	+0.33	+7.6	−0.43 to +1.09	0.330	+0.28		
Functional MVC sEMG (µV)												
	Right temporalis	Orthosis	36	122.21 ± 11.68	137.70 ± 17.50	+15.49	+12.7	+8.07 to +22.92	<0.001	+0.71	0.694	−0.08
		Observation	12	119.88 ± 10.77	136.97 ± 7.37	+17.08	+14.2	+9.15 to +25.02	<0.001	+1.37		
	Left temporalis	Orthosis	36	127.74 ± 11.81	160.89 ± 34.72	+33.15	+26.0	+22.43 to +43.88	<0.001	+1.05	0.002	+0.60
		Observation	12	134.11 ± 12.32	138.12 ± 9.04	+4.02	+3.0	−6.68 to +14.72	0.677	+0.24		
	Right masseter	Orthosis	36	136.47 ± 12.68	165.91 ± 30.79	+29.45	+21.6	+19.24 to +39.66	<0.001	+0.98	<0.001	+0.87
		Observation	12	140.87 ± 8.79	131.53 ± 9.67	−9.34	−6.6	−16.61 to −2.08	0.016	−0.82		
	Left masseter	Orthosis	36	139.84 ± 12.12	167.94 ± 34.97	+28.09	+20.1	+16.54 to +39.64	<0.001	+0.82	<0.001	+0.76
		Observation	12	138.95 ± 8.21	135.80 ± 7.82	−3.15	−2.3	−10.43 to +4.13	0.206	−0.27		

*Positive functional changes and negative resting changes in the orthosis cohort support the composite interpretation of improved neuromuscular efficiency. Values are reported as mean ± standard deviation. sEMG, surface electromyography; MVC, maximal voluntary clenching; CI, confidence interval; Std., standardized. Mean change was calculated as 3 months minus baseline. Negative changes in resting sEMG and positive changes in functional MVC sEMG were considered favorable. Within-group p values were calculated using Wilcoxon signed-rank tests. Between-group p values compare change scores between cohorts using Mann-Whitney U tests. Standardized effect was calculated as mean paired change divided by the standard deviation of the paired change. Cliff delta compares orthosis versus observation change scores.*

**Fig. 4.** Composite surface electromyographic (sEMG) activity at baseline and 3-month follow-up. (A) Resting composite sEMG and (B) functional composite sEMG in the orthosis and observation cohorts at baseline and after approximately 3 months. Bars represent mean values and error bars indicate 95% confidence intervals. The orthosis cohort showed reduced resting sEMG activity and increased functional recruitment, whereas the observation cohort showed the opposite trend.

Composite resting sEMG activity decreased significantly following orthopedic stabilization, whereas the observation cohort demonstrated increased resting activity over the same period. Conversely, composite functional sEMG activity during maximal voluntary clenching increased significantly in the orthopedic stabilization cohort but remained essentially unchanged in the observation cohort. Significant between-group differences were identified for both resting and functional recording conditions.

Analysis of individual muscles demonstrated a consistent reduction in resting activity following orthopedic stabilization, particularly in the anterior temporalis muscles and the left masseter. During maximal voluntary clenching, all evaluated muscles demonstrated increased functional recruitment in the orthopedic stabilization cohort. In contrast, the observation cohort showed increased resting temporalis activity without a comparable increase in functional muscle recruitment.

### 3.4 SPECT/CT findings

Secondary SPECT/CT outcomes are summarized in Table 4.

**Table 4.** Secondary SPECT/CT metabolic outcomes.

Outcome	Group	n	Baseline	Final follow-up	Mean change	Change (%)	95% CI for change	Within-group *p*	Std. effect	Between-group *p*	Cliff delta
Coronal SPECT/CT uptake/activity ratio											
	Orthosis	37	1.29 ± 0.18	0.61 ± 0.29	−0.67	−52.3	−0.77 to −0.58	<0.001	−2.33	<0.001	−0.95
	Observation	12	1.41 ± 0.09	1.62 ± 0.08	+0.22	+15.4	+0.14 to +0.29	<0.001	+1.82		
Axial SPECT/CT uptake/activity ratio											
	Orthosis	37	1.26 ± 0.33	0.93 ± 0.26	−0.33	−26.4	−0.49 to −0.18	<0.001	−0.71	<0.001	−0.88
	Observation	12	1.22 ± 0.10	1.70 ± 0.11	+0.48	+38.8	+0.39 to +0.56	<0.001	+3.69		

*These semiquantitative uptake/activity ratios should be interpreted as secondary metabolic observations and not as evidence of condylar growth arrest. Values are reported as mean ± standard deviation. SPECT/CT, single-photon emission computed tomography/computed tomography; CI, confidence interval; Std., standardized. Mean change was calculated as final follow-up minus baseline. Within-group p values were calculated using Wilcoxon signed-rank tests. Between-group p values compare change scores between cohorts using Mann-Whitney U tests. Standardized effect was calculated as mean paired change divided by the standard deviation of the paired change. Cliff delta compares orthosis versus observation change scores; negative values indicate larger decreases in the orthosis cohort.*

Coronal and axial condylar uptake ratios decreased significantly during follow-up in the orthopedic stabilization cohort, whereas the observation cohort demonstrated increased uptake over the same period. Significant between-group differences were identified for both imaging planes (Table 4).

Representative CBCT images are presented in Fig. 5 to illustrate the imaging characteristics of active unilateral condylar hyperplasia. These images are provided for illustrative purposes only and were not included in the quantitative analyses. As described in the Methods, SPECT/CT findings should be interpreted as semiquantitative indicators of relative osteoblastic activity rather than as direct measures of condylar growth or biological growth arrest.

**Fig. 5.** Representative CBCT images of active unilateral left condylar hyperplasia. Bilateral sagittal and coronal reconstructions demonstrate condylar asymmetry. The coronal reference lines illustrate the relationship between each glenoid fossa and a true horizontal plane, highlighting the adaptive remodeling of the left glenoid fossa associated with condylar hyperplasia.

## 4. Discussion

The most relevant finding of the present investigation was not the metabolic variation observed in SPECT/CT examinations, but the consistent pattern of clinical and neuromuscular adaptation documented following full-time orthopedic stabilization in patients with active condylar hyperplasia. Improvements in mandibular symmetry, mouth opening, and electromyographic behavior, together with descriptively favorable pain findings, are consistent with the hypothesis that active condylar hyperplasia affects the entire stomatognathic system and that functional stabilization may contribute to this adaptive response. These observations complement previous investigations addressing functional adaptations associated with stabilization therapy [28], as well as studies primarily focused on condylar growth activity and surgical management in condylar hyperplasia [29].

The observed reduction in mandibular midline deviation may be clinically relevant because facial asymmetry represents one of the most visible manifestations of active unilateral condylar hyperplasia. Although the present study was not designed to establish a minimal clinically important difference, a mean reduction of approximately one third of the initial deviation was accompanied by improvements in mandibular centering and functional parameters, suggesting that the observed changes may be perceptible both clinically and aesthetically.

Although the absolute increase in maximum mouth opening was relatively modest, it should be interpreted together with the concomitant reduction in mandibular midline deviation, improved electromyographic findings, and descriptively favorable pain evolution. Considered collectively rather than as isolated variables, these findings suggest a clinically relevant pattern of functional adaptation during orthopedic stabilization.

The present findings are consistent with the concept that active condylar hyperplasia represents a biologically heterogeneous condition requiring individualized assessment and treatment planning. Wolford and colleagues emphasized the importance of identifying active growth before therapeutic decision-making, a concept that has recently been reinforced by contemporary clinical practice guidelines and expert consensus recommendations [2, 12, 13]. Our findings do not challenge these concepts; rather, they extend them by demonstrating that clinically measurable adaptation may occur during the period preceding definitive treatment. Unlike previous studies focused primarily on growth progression or surgical correction, the present investigation evaluated functional behavior during observation and stabilization.

The reduction in resting electromyographic activity observed after stabilization is consistent with physiological patterns reported in healthy individuals, where low resting muscle activity has been associated with neuromuscular balance, muscular coordination, and efficient stomatognathic function, as described by Ferrario and Sforza [30, 31, 32, 33]. In healthy young adults, resting sEMG values of approximately 1.4 μV for the masseter and 1.9 μV for the anterior temporalis have been reported as physiological reference values [34]. Although these reference values were not used as diagnostic thresholds in the present study, they provide a useful biological framework for interpreting the reduction in resting muscle activity observed after orthopedic stabilization. Simultaneously, the increase in functional muscle recruitment differs from the pattern expected in generalized muscular inhibition. Likewise, the higher EMG amplitudes observed during maximal voluntary clenching moved toward the physiological pattern of muscle activation described in healthy individuals, where substantially greater muscle recruitment is expected during maximal contraction than at rest. This interpretation is further supported by the concomitant improvements observed in mandibular function and facial symmetry in the treated cohort; however, these findings should be interpreted within the limitations inherent to the observational design. Therefore, our findings are consistent with the physiological concepts proposed by Ferrario and Sforza [30, 31, 32, 33], while extending these observations to patients with active unilateral condylar hyperplasia.

Beyond these physiological reference values, the present findings are also consistent with contemporary recommendations indicating that the overall pattern of muscle activity and longitudinal within-subject changes are generally more informative than isolated absolute EMG values [35]. Accordingly, the electromyographic findings should be interpreted within the contemporary framework proposed by Castroflorio *et al*. [35, 36], and further supported by Zieliński and Gawda [37], emphasizing that surface electromyography is best regarded as a functional assessment tool rather than a diagnostic test. Our findings are consistent with this interpretation because sEMG was used to document adaptive changes over time rather than to establish a specific diagnosis or demonstrate treatment efficacy on the basis of electromyographic findings alone. Consequently, the present findings should not be interpreted as evidence of an ideal condylar position, but rather as indicators of functional adaptation occurring during orthopedic stabilization. Accordingly, sEMG should be interpreted as a complementary functional outcome measure within a comprehensive clinical assessment rather than as an isolated diagnostic modality. This approach is particularly appropriate in longitudinal studies designed to evaluate functional adaptation rather than to establish a diagnosis.

Pain intensity was recorded as a clinical outcome using a visual analog scale; however, paired baseline and follow-up pain scores were not available for all participants. Consequently, pain findings could only be summarized descriptively and should not be interpreted as evidence of longitudinal treatment-related change. Nevertheless, the descriptively favorable pain findings observed in the treated cohort are generally consistent with reports describing symptomatic improvement following stabilization of the stomatognathic system [16, 17, 18, 19, 20]. Direct comparisons should be interpreted cautiously because most previous investigations evaluated patients with temporomandibular disorders rather than active unilateral condylar hyperplasia. Unlike conventional temporomandibular disorders, active unilateral condylar hyperplasia represents an active growth condition capable of generating progressive asymmetry. Therefore, the reduction in mandibular deviation observed in the present study may have particular clinical relevance in this specific patient population.

Previous studies have consistently demonstrated the value of SPECT/CT for identifying active condylar growth and supporting treatment planning in patients with unilateral condylar hyperplasia [7, 8, 9, 10]. Our findings do not contradict those observations, but suggest that metabolic findings should be interpreted together with the accompanying clinical and functional adaptations. Unlike investigations in which metabolic activity constituted the primary outcome, the present study considered SPECT/CT findings as a complementary outcome measure. Consequently, changes in metabolic activity should not be interpreted as definitive evidence of growth modification, but rather as semiquantitative observations accompanying the broader biological response documented clinically and electromyographically.

Orthopedic stabilization is not intended to suppress condylar growth, but rather to optimize the neuromuscular environment in which mandibular function occurs, thereby facilitating functional adaptation despite the persistence of active condylar metabolic activity.

An additional aspect deserving consideration is the potential influence of functional stabilization on the contralateral temporomandibular joint, as asymmetrical growth patterns and adaptive remodeling have been previously described in patients with unilateral condylar hyperplasia [38, 39, 40]. In this context, the reduction in mandibular deviation and the improvement in neuromuscular balance observed in the present cohort are consistent with the hypothesis that functional stabilization may contribute to a more symmetrical biomechanical environment. This hypothesis may be particularly relevant in patients presenting adaptive remodeling or progressive resorptive changes in the contralateral joint. Nevertheless, because intra-articular loading was not directly measured, this hypothesis should be regarded as hypothesis-generating rather than confirmatory and requires further investigation using direct biomechanical and imaging assessments.

The present study has several strengths, including the use of a comparative cohort, the simultaneous evaluation of clinical, electromyographic, and metabolic outcomes, and the inclusion of patients with active unilateral condylar hyperplasia confirmed by SPECT/CT. Nevertheless, several limitations should also be acknowledged. The absence of random allocation limits causal inference, the sample size remains moderate, follow-up duration does not permit conclusions regarding long-term condylar growth behavior, and direct measurements of intra-articular loading were not available. Furthermore, although the observational cohort strengthened the comparative analyses, treatment allocation reflected routine clinical decision-making rather than experimental assignment.

Taken together, these findings are consistent with a broader biological perspective in which active unilateral condylar hyperplasia is understood not only as a disorder of condylar growth, but also as a condition capable of inducing adaptive responses throughout the stomatognathic system. Functional stabilization should not be interpreted as a substitute for definitive treatment when such treatment is indicated. Rather, it may provide conditions that facilitate functional adaptation and a potentially more favorable biomechanical environment. This interpretation should be regarded as a biologically plausible hypothesis rather than an established mechanism. A conceptual summary of the proposed biological model is presented in Fig. 6.

**Fig. 6.** Proposed conceptual pathway linking orthopedic stabilization to the observed functional and imaging changes in patients with active condylar hyperplasia. Full-time orthopedic stabilization provides a reproducible mandibular position, improving mandibular centering and reducing asymmetric joint loading. Repeated function under these more stable biomechanical conditions may facilitate neuromuscular adaptation, characterized by reduced resting muscle activity and more efficient functional recruitment on sEMG. These functional changes may contribute to a more balanced biomechanical environment, with secondary adaptive metabolic findings observed on SPECT/CT. The sequence represents a biologically plausible mechanistic hypothesis based on the clinical, electromyographic, and imaging findings of the present study and should not be interpreted as evidence of direct causality. SPECT/CT, single-photon emission computed tomography/computed tomography.

## 5. Limitations

This study has several limitations. First, the design was observational and non-randomized; therefore, causal inference must be interpreted with caution. Second, the treated and observation cohorts were not created by random allocation and may differ in unmeasured clinical factors. Although treatment allocation was not based on disease severity per se, unmeasured clinical factors may have influenced the decision to initiate orthopedic stabilization or observation. Despite this inherent limitation of the observational design, baseline demographic, clinical, and neuromuscular characteristics were generally comparable between cohorts, suggesting that major differences in initial disease presentation were unlikely to account for the observed functional outcomes. Nevertheless, the possibility of residual selection bias cannot be completely excluded. In addition, the relatively small size of the observation cohort may have reduced statistical power for some between-group comparisons, particularly when interpreting nonsignificant findings. Third, sEMG was evaluated as an exploratory functional outcome rather than as a diagnostic measure. Fourth, clinical, sEMG, and SPECT/CT outcomes were assessed over different time scales because they reflect different biological processes: early neuromuscular adaptation was assessed after approximately 3 months, whereas metabolic activity was reassessed after a longer mean follow-up of approximately 14 months. In addition, paired baseline and follow-up pain scores were not available for all participants; therefore, pain intensity could only be analyzed descriptively rather than as a longitudinal outcome. Fifth, the study did not include standardized quality-of-life instruments, direct joint loading measurements, serial magnetic resonance imaging (MRI) of the contralateral joint, or phenotype-specific stratification according to hemimandibular elongation, hemimandibular hyperplasia, or mixed forms. Finally, a formal a priori sample size calculation was not feasible because active unilateral condylar hyperplasia is an uncommon condition, and the study was designed as a prospective observational cohort including all consecutive eligible patients treated during the recruitment period. Consequently, the sample was determined by the number of consecutive patients meeting the predefined inclusion criteria rather than by a predetermined statistical target. Therefore, the findings should be confirmed in larger, prospectively powered studies.

## 6. Conclusions

Full-time orthopedic functional stabilization was associated with measurable clinical and neuromuscular adaptation during the observation period in patients with active unilateral condylar hyperplasia. Patients managed with orthopedic stabilization demonstrated improved mandibular midline deviation, increased maximum mouth opening, lower resting sEMG activity, and greater functional muscle recruitment compared with those managed by observation alone.

These findings support the hypothesis that orthopedic functional stabilization promotes functional and neuromuscular adaptation within the stomatognathic system during the observation period.

SPECT/CT metabolic findings should be interpreted as complementary semiquantitative observations rather than as evidence of condylar growth modification or biological growth arrest.

The hypothesis that orthopedic functional stabilization may contribute to a more favorable biomechanical environment, including the contralateral temporomandibular joint, is biologically plausible but requires confirmation through adequately powered prospective studies incorporating direct imaging and biomechanical assessment.
